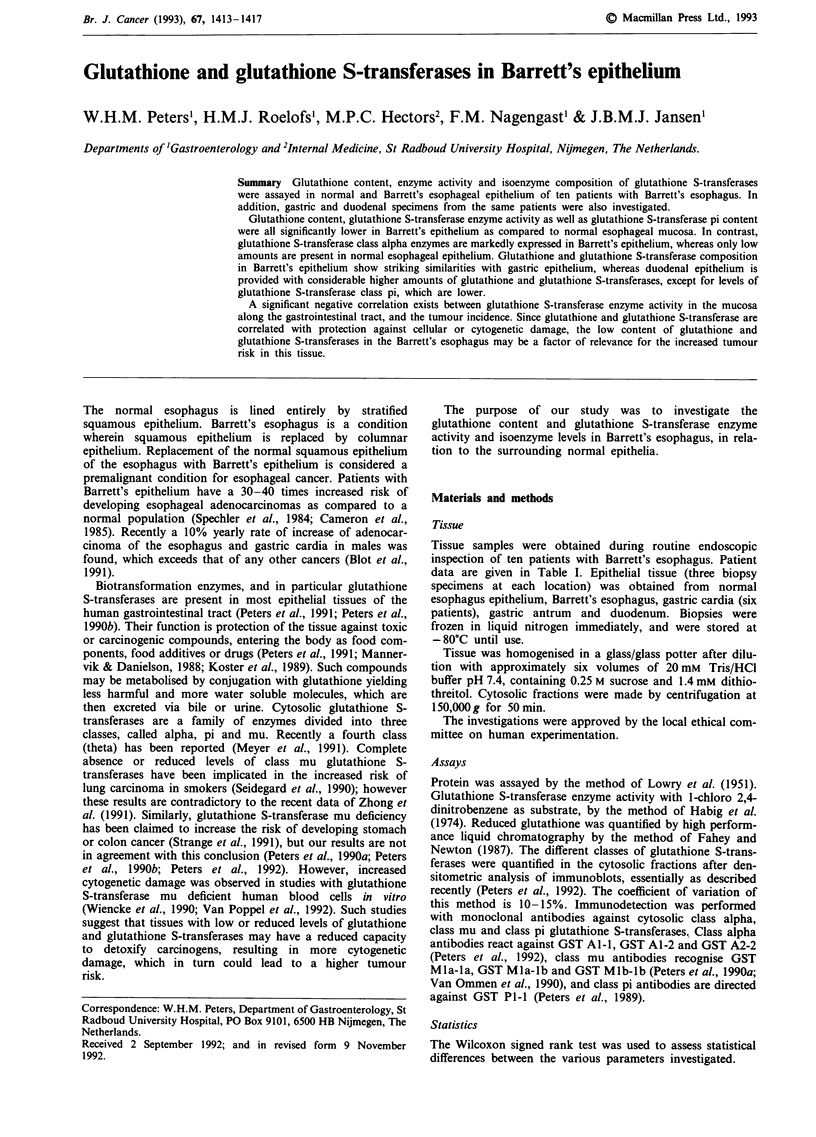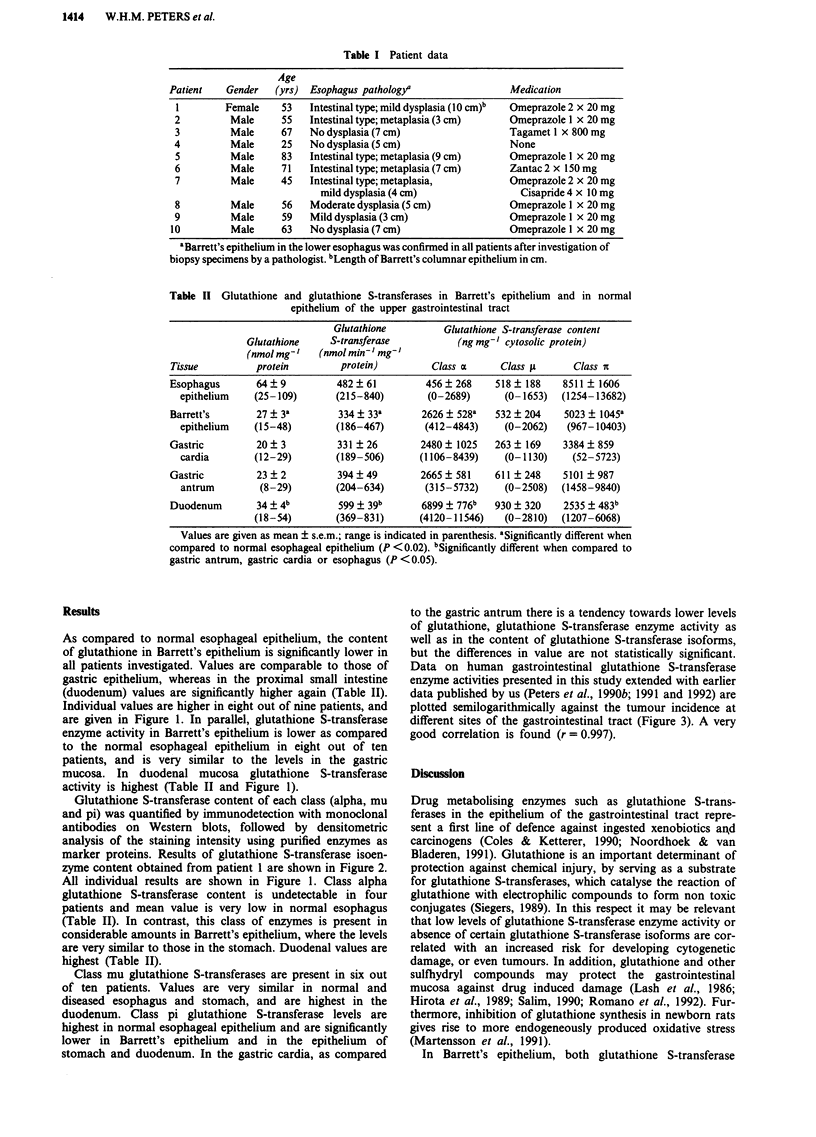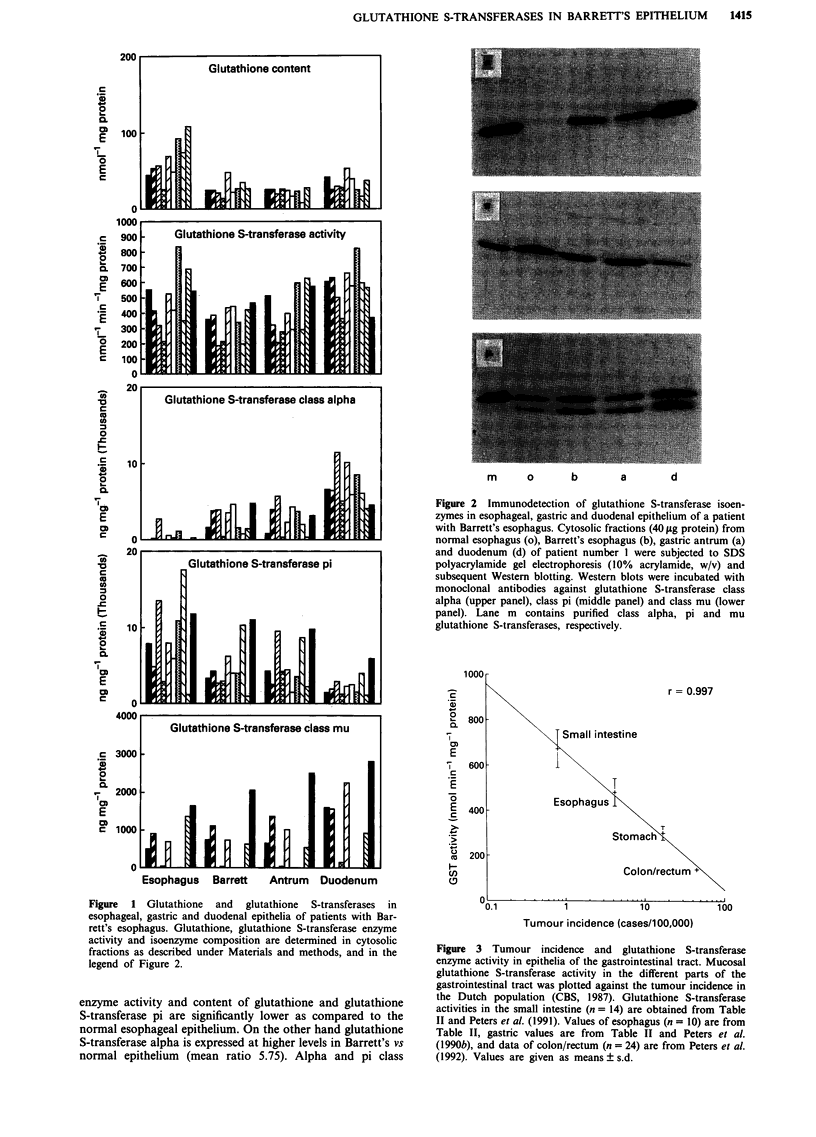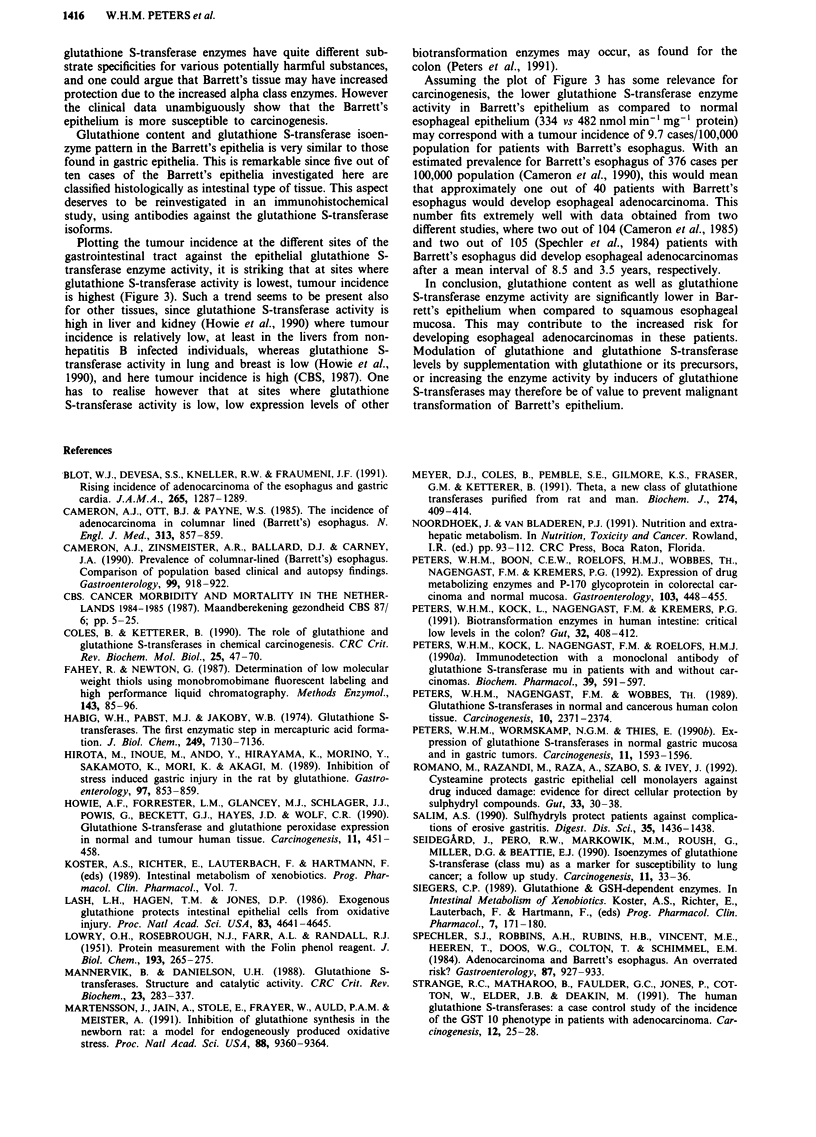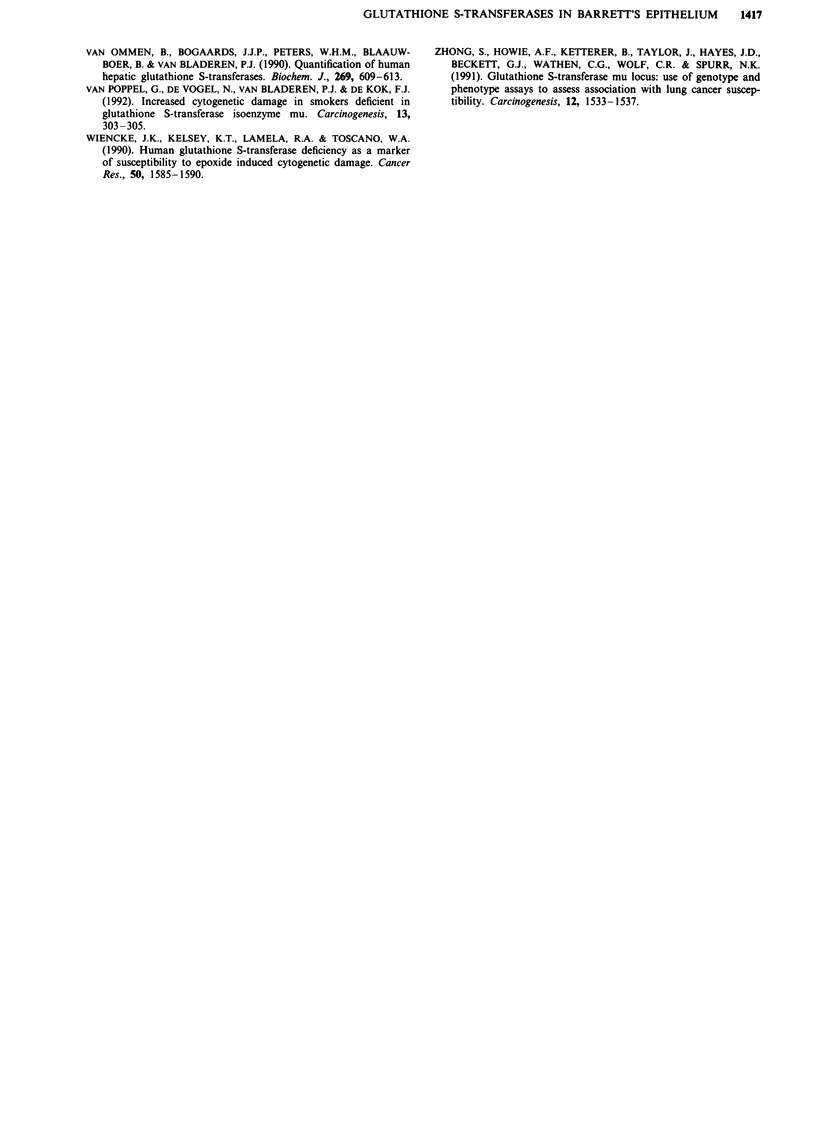# Glutathione and glutathione S-transferases in Barrett's epithelium.

**DOI:** 10.1038/bjc.1993.262

**Published:** 1993-06

**Authors:** W. H. Peters, H. M. Roelofs, M. P. Hectors, F. M. Nagengast, J. B. Jansen

**Affiliations:** Department of Gastroenterology, St Radboud University Hospital, Nijmegen, The Netherlands.

## Abstract

**Images:**


*Br. J. Cancer* (1993), **67**, 1413–1417 © Macmillan Press Ltd., 1993

### Glutathione and glutathione S-transferases in Barrett's epithelium

W.H.M. Peters[1], H.M.J. Roelofs[1], M.P.C. Hectors[2], F.M. Nagengast[1] & J.B.M.J. Jansen[1]

*Departments of [1]Gastroenterology and [2]Internal Medicine, St Radboud University Hospital, Nijmegen, The Netherlands.*

**Summary** Glutathione content, enzyme activity and isoenzyme composition of glutathione S-transferases were assayed in normal and Barrett's esophageal epithelium of ten patients with Barrett's esophagus. In addition, gastric and duodenal specimens from the same patients were also investigated.

Glutathione content, glutathione S-transferase enzyme activity as well as glutathione S-transferase pi content were all significantly lower in Barrett's epithelium as compared to normal esophageal mucosa. In contrast, glutathione S-transferase class alpha enzymes are markedly expressed in Barrett's epithelium, whereas only low amounts are present in normal esophageal epithelium. Glutathione and glutathione S-transferase composition in Barrett's epithelium show striking similarities with gastric epithelium, whereas duodenal epithelium is provided with considerable higher amounts of glutathione and glutathione S-transferases, except for levels of glutathione S-transferase class pi, which are lower.

A significant negative correlation exists between glutathione S-transferase enzyme activity in the mucosa along the gastrointestinal tract, and the tumour incidence. Since glutathione and glutathione S-transferase are correlated with protection against cellular or cytogenetic damage, the low content of glutathione and glutathione S-transferases in the Barrett's esophagus may be a factor of relevance for the increased tumour risk in this tissue.

The normal esophagus is lined entirely by stratified squamous epithelium. Barrett's esophagus is a condition wherein squamous epithelium is replaced by columnar epithelium. Replacement of the normal squamous epithelium of the esophagus with Barrett's epithelium is considered a premalignant condition for esophageal cancer. Patients with Barrett's epithelium have a 30–40 times increased risk of developing esophageal adenocarcinomas as compared to a normal population (Spechler *et al.*, 1984; Cameron *et al.*, 1985). Recently a 10% yearly rate of increase of adenocarcinoma of the esophagus and gastric cardia in males was found, which exceeds that of any other cancers (Blot *et al.*, 1991).

Biotransformation enzymes, and in particular glutathione S-transferases are present in most epithelial tissues of the human gastrointestinal tract (Peters *et al.*, 1991; Peters *et al.*, 1990*b*). Their function is protection of the tissue against toxic or carcinogenic compounds, entering the body as food components, food additives or drugs (Peters *et al.*, 1991; Mannervik & Danielson, 1988; Koster *et al.*, 1989). Such compounds may be metabolised by conjugation with glutathione yielding less harmful and more water soluble molecules, which are then excreted via bile or urine. Cytosolic glutathione S-transferases are a family of enzymes divided into three classes, called alpha, pi and mu. Recently a fourth class (theta) has been reported (Meyer *et al.*, 1991). Complete absence or reduced levels of class mu glutathione S-transferases have been implicated in the increased risk of lung carcinoma in smokers (Seidegard *et al.*, 1990); however these results are contradictory to the recent data of Zhong *et al.* (1991). Similarly, glutathione S-transferase mu deficiency has been claimed to increase the risk of developing stomach or colon cancer (Strange *et al.*, 1991), but our results are not in agreement with this conclusion (Peters *et al.*, 1990*a*; Peters *et al.*, 1990*b*; Peters *et al.*, 1992). However, increased cytogenetic damage was observed in studies with glutathione S-transferase mu deficient human blood cells *in vitro* (Wiencke *et al.*, 1990; Van Poppel *et al.*, 1992). Such studies suggest that tissues with low or reduced levels of glutathione and glutathione S-transferases may have a reduced capacity to detoxify carcinogens, resulting in more cytogenetic damage, which in turn could lead to a higher tumour risk.

Correspondence: W.H.M. Peters, Department of Gastroenterology, St Radboud University Hospital, PO Box 9101, 6500 HB Nijmegen, The Netherlands.

Received 2 September 1992; and in revised form 9 November 1992.

The purpose of our study was to investigate the glutathione content and glutathione S-transferase enzyme activity and isoenzyme levels in Barrett's esophagus, in relation to the surrounding normal epithelia.

### Materials and methods

### *Tissue*

Tissue samples were obtained during routine endoscopic inspection of ten patients with Barrett's esophagus. Patient data are given in Table I. Epithelial tissue (three biopsy specimens at each location) was obtained from normal esophagus epithelium, Barrett's esophagus, gastric cardia (six patients), gastric antrum and duodenum. Biopsies were frozen in liquid nitrogen immediately, and were stored at −80°C until use.

Tissue was homogenised in a glass/glass potter after dilution with approximately six volumes of 20 mM Tris/HCl buffer pH 7.4, containing 0.25 M sucrose and 1.4 mM dithiothreitol. Cytosolic fractions were made by centrifugation at 150,000 *g* for 50 min.

The investigations were approved by the local ethical committee on human experimentation.

### *Assays*

Protein was assayed by the method of Lowry *et al.* (1951). Glutathione S-transferase enzyme activity with 1-chloro 2,4-dinitrobenzene as substrate, by the method of Habig *et al.* (1974). Reduced glutathione was quantified by high performance liquid chromatography by the method of Fahey and Newton (1987). The different classes of glutathione S-transferases were quantified in the cytosolic fractions after densitometric analysis of immunoblots, essentially as described recently (Peters *et al.*, 1992). The coefficient of variation of this method is 10–15%. Immunodetection was performed with monoclonal antibodies against cytosolic class alpha, class mu and class pi glutathione S-transferases. Class alpha antibodies react against GST A1-1, GST A1-2 and GST A2-2 (Peters *et al.*, 1992), class mu antibodies recognise GST M1a-1a, GST M1a-1b and GST M1b-1b (Peters *et al.*, 1990*a*; Van Ommen *et al.*, 1990), and class pi antibodies are directed against GST P1-1 (Peters *et al.*, 1989).

### *Statistics*

The Wilcoxon signed rank test was used to assess statistical differences between the various parameters investigated.

**Table I** Patient data

| Patient | Gender | Age (yrs) | Esophagus pathology[a] | Medication |
|---|---|---|---|---|
| 1 | Female | 53 | Intestinal type; mild dysplasia (10 cm)[b] | Omeprazole 2 × 20 mg |
| 2 | Male | 55 | Intestinal type; metaplasia (3 cm) | Omeprazole 1 × 20 mg |
| 3 | Male | 67 | No dysplasia (7 cm) | Tagamet 1 × 800 mg |
| 4 | Male | 25 | No dysplasia (5 cm) | None |
| 5 | Male | 83 | Intestinal type; metaplasia (9 cm) | Omeprazole 1 × 20 mg |
| 6 | Male | 71 | Intestinal type; metaplasia (7 cm) | Zantac 2 × 150 mg |
| 7 | Male | 45 | Intestinal type; metaplasia, mild dysplasia (4 cm) | Omeprazole 2 × 20 mg Cisapride 4 × 10 mg |
| 8 | Male | 56 | Moderate dysplasia (5 cm) | Omeprazole 1 × 20 mg |
| 9 | Male | 59 | Mild dysplasia (3 cm) | Omeprazole 1 × 20 mg |
| 10 | Male | 63 | No dysplasia (7 cm) | Omeprazole 1 × 20 mg |

[a]Barrett's epithelium in the lower esophagus was confirmed in all patients after investigation of biopsy specimens by a pathologist. [b]Length of Barrett's columnar epithelium in cm.

**Table II** Glutathione and glutathione S-transferases in Barrett's epithelium and in normal epithelium of the upper gastrointestinal tract

| Tissue | Glutathione (nmol mg$^{-1}$ protein | Glutathione S-transferase (nmol min$^{-1}$ mg$^{-1}$ protein) | Glutathione S-transferase content (ng mg$^{-1}$ cytosolic protein) Class α | Class μ | Class π |
|---|---|---|---|---|---|
| Esophagus epithelium | 64 ± 9 (25–109) | 482 ± 61 (215–840) | 456 ± 268 (0–2689) | 518 ± 188 (0–1653) | 8511 ± 1606 (1254–13682) |
| Barrett's epithelium | 27 ± 3[a] (15–48) | 334 ± 33[a] (186–467) | 2626 ± 528[a] (412–4843) | 532 ± 204 (0–2062) | 5023 ± 1045[a] (967–10403) |
| Gastric cardia | 20 ± 3 (12–29) | 331 ± 26 (189–506) | 2480 ± 1025 (1106–8439) | 263 ± 169 (0–1130) | 3384 ± 859 (52–5723) |
| Gastric antrum | 23 ± 2 (8–29) | 394 ± 49 (204–634) | 2665 ± 581 (315–5732) | 611 ± 248 (0–2508) | 5101 ± 987 (1458–9840) |
| Duodenum | 34 ± 4[b] (18–54) | 599 ± 39[b] (369–831) | 6899 ± 776[b] (4120–11546) | 930 ± 320 (0–2810) | 2535 ± 483[b] (1207–6068) |

Values are given as mean ± s.e.m.; range is indicated in parenthesis. [a]Significantly different when compared to normal esophageal epithelium ($P < 0.02$). [b]Significantly different when compared to gastric antrum, gastric cardia or esophagus ($P < 0.05$).

### Results

As compared to normal esophageal epithelium, the content of glutathione in Barrett's epithelium is significantly lower in all patients investigated. Values are comparable to those of gastric epithelium, whereas in the proximal small intestine (duodenum) values are significantly higher again (Table II). Individual values are higher in eight out of nine patients, and are given in Figure 1. In parallel, glutathione S-transferase enzyme activity in Barrett's epithelium is lower as compared to the normal esophageal epithelium in eight out of ten patients, and is very similar to the levels in the gastric mucosa. In duodenal mucosa glutathione S-transferase activity is highest (Table II and Figure 1).

Glutathione S-transferase content of each class (alpha, mu and pi) was quantified by immunodetection with monoclonal antibodies on Western blots, followed by densitometric analysis of the staining intensity using purified enzymes as marker proteins. Results of glutathione S-transferase isoenzyme content obtained from patient 1 are shown in Figure 2. All individual results are shown in Figure 1. Class alpha glutathione S-transferase content is undetectable in four patients and mean value is very low in normal esophagus (Table II). In contrast, this class of enzymes is present in considerable amounts in Barrett's epithelium, where the levels are very similar to those in the stomach. Duodenal values are highest (Table II).

Class mu glutathione S-transferases are present in six out of ten patients. Values are very similar in normal and diseased esophagus and stomach, and are highest in the duodenum. Class pi glutathione S-transferase levels are highest in normal esophageal epithelium and are significantly lower in Barrett's epithelium and in the epithelium of stomach and duodenum. In the gastric cardia, as compared to the gastric antrum there is a tendency towards lower levels of glutathione, glutathione S-transferase enzyme activity as well as in the content of glutathione S-transferase isoforms, but the differences in value are not statistically significant. Data on human gastrointestinal glutathione S-transferase enzyme activities presented in this study extended with earlier data published by us (Peters et al., 1990b; 1991 and 1992) are plotted semilogarithmically against the tumour incidence at different sites of the gastrointestinal tract (Figure 3). A very good correlation is found ($r = 0.997$).

### Discussion

Drug metabolising enzymes such as glutathione S-transferases in the epithelium of the gastrointestinal tract represent a first line of defence against ingested xenobiotics and carcinogens (Coles & Ketterer, 1990; Noordhoek & van Bladeren, 1991). Glutathione is an important determinant of protection against chemical injury, by serving as a substrate for glutathione S-transferases, which catalyse the reaction of glutathione with electrophilic compounds to form non toxic conjugates (Siegers, 1989). In this respect it may be relevant that low levels of glutathione S-transferase enzyme activity or absence of certain glutathione S-transferase isoforms are correlated with an increased risk for developing cytogenetic damage, or even tumours. In addition, glutathione and other sulfhydryl compounds may protect the gastrointestinal mucosa against drug induced damage (Lash et al., 1986; Hirota et al., 1989; Salim, 1990; Romano et al., 1992). Furthermore, inhibition of glutathione synthesis in newborn rats gives rise to more endogeneously produced oxidative stress (Martensson et al., 1991).

In Barrett's epithelium, both glutathione S-transferase

**Figure 1** Glutathione and glutathione S-transferases in esophageal, gastric and duodenal epithelia of patients with Barrett's esophagus. Glutathione, glutathione S-transferase enzyme activity and isoenzyme composition are determined in cytosolic fractions as described under Materials and methods, and in the legend of Figure 2.

**Figure 2** Immunodetection of glutathione S-transferase isoenzymes in esophageal, gastric and duodenal epithelium of a patient with Barrett's esophagus. Cytosolic fractions (40 μg protein) from normal esophagus (o), Barrett's esophagus (b), gastric antrum (a) and duodenum (d) of patient number 1 were subjected to SDS polyacrylamide gel electrophoresis (10% acrylamide, w/v) and subsequent Western blotting. Western blots were incubated with monoclonal antibodies against glutathione S-transferase class alpha (upper panel), class pi (middle panel) and class mu (lower panel). Lane m contains purified class alpha, pi and mu glutathione S-transferases, respectively.

**Figure 3** Tumour incidence and glutathione S-transferase enzyme activity in epithelia of the gastrointestinal tract. Mucosal glutathione S-transferase activity in the different parts of the gastrointestinal tract was plotted against the tumour incidence in the Dutch population (CBS, 1987). Glutathione S-transferase activities in the small intestine ($n = 14$) are obtained from Table II and Peters *et al.* (1991). Values of esophagus ($n = 10$) are from Table II, gastric values are from Table II and Peters *et al.* (1990*b*), and data of colon/rectum ($n = 24$) are from Peters *et al.* (1992). Values are given as means ± s.d.

enzyme activity and content of glutathione and glutathione S-transferase pi are significantly lower as compared to the normal esophageal epithelium. On the other hand glutathione S-transferase alpha is expressed at higher levels in Barrett's *vs* normal epithelium (mean ratio 5.75). Alpha and pi class glutathione S-transferase enzymes have quite different substrate specificities for various potentially harmful substances, and one could argue that Barrett's tissue may have increased protection due to the increased alpha class enzymes. However the clinical data unambiguously show that the Barrett's epithelium is more susceptible to carcinogenesis.

Glutathione content and glutathione S-transferase isoenzyme pattern in the Barrett's epithelia is very similar to those found in gastric epithelia. This is remarkable since five out of ten cases of the Barrett's epithelia investigated here are classified histologically as intestinal type of tissue. This aspect deserves to be reinvestigated in an immunohistochemical study, using antibodies against the glutathione S-transferase isoforms.

Plotting the tumour incidence at the different sites of the gastrointestinal tract against the epithelial glutathione S-transferase enzyme activity, it is striking that at sites where glutathione S-transferase activity is lowest, tumour incidence is highest (Figure 3). Such a trend seems to be present also for other tissues, since glutathione S-transferase activity is high in liver and kidney (Howie *et al.*, 1990) where tumour incidence is relatively low, at least in the livers from non-hepatitis B infected individuals, whereas glutathione S-transferase activity in lung and breast is low (Howie *et al.*, 1990), and here tumour incidence is high (CBS, 1987). One has to realise however that at sites where glutathione S-transferase activity is low, low expression levels of other biotransformation enzymes may occur, as found for the colon (Peters *et al.*, 1991).

Assuming the plot of Figure 3 has some relevance for carcinogenesis, the lower glutathione S-transferase enzyme activity in Barrett's epithelium as compared to normal esophageal epithelium (334 *vs* 482 nmol $min^{-1}$ $mg^{-1}$ protein) may correspond with a tumour incidence of 9.7 cases/100,000 population for patients with Barrett's esophagus. With an estimated prevalence for Barrett's esophagus of 376 cases per 100,000 population (Cameron *et al.*, 1990), this would mean that approximately one out of 40 patients with Barrett's esophagus would develop esophageal adenocarcinoma. This number fits extremely well with data obtained from two different studies, where two out of 104 (Cameron *et al.*, 1985) and two out of 105 (Spechler *et al.*, 1984) patients with Barrett's esophagus did develop esophageal adenocarcinomas after a mean interval of 8.5 and 3.5 years, respectively.

In conclusion, glutathione content as well as glutathione S-transferase enzyme activity are significantly lower in Barrett's epithelium when compared to squamous esophageal mucosa. This may contribute to the increased risk for developing esophageal adenocarcinomas in these patients. Modulation of glutathione and glutathione S-transferase levels by supplementation with glutathione or its precursors, or increasing the enzyme activity by inducers of glutathione S-transferases may therefore be of value to prevent malignant transformation of Barrett's epithelium.

### References

BLOT, W.J., DEVESA, S.S., KNELLER, R.W. & FRAUMENI, J.F. (1991). Rising incidence of adenocarcinoma of the esophagus and gastric cardia. *J.A.M.A.*, **265**, 1287–1289.

CAMERON, A.J., OTT, B.J. & PAYNE, W.S. (1985). The incidence of adenocarcinoma in columnar lined (Barrett's) esophagus. *N. Engl. J. Med.*, **313**, 857–859.

CAMERON, A.J., ZINSMEISTER, A.R., BALLARD, D.J. & CARNEY, J.A. (1990). Prevalence of columnar-lined (Barrett's) esophagus. Comparison of population based clinical and autopsy findings. *Gastroenterology*, **99**, 918–922.

CBS. CANCER MORBIDITY AND MORTALITY IN THE NETHERLANDS 1984–1985 (1987). Maandberekening gezondheid CBS 87/6; pp. 5–25.

COLES, B. & KETTERER, B. (1990). The role of glutathione and glutathione S-transferases in chemical carcinogenesis. *CRC Crit. Rev. Biochem. Mol. Biol.*, **25**, 47–70.

FAHEY, R. & NEWTON, G. (1987). Determination of low molecular weight thiols using monobromobimane fluorescent labeling and high performance liquid chromatography. *Methods Enzymol.*, **143**, 85–96.

HABIG, W.H., PABST, M.J. & JAKOBY, W.B. (1974). Glutathione S-transferases. The first enzymatic step in mercapturic acid formation. *J. Biol. Chem.*, **249**, 7130–7136.

HIROTA, M., INOUE, M., ANDO, Y., HIRAYAMA, K., MORINO, Y., SAKAMOTO, K., MORI, K. & AKAGI, M. (1989). Inhibition of stress induced gastric injury in the rat by glutathione. *Gastroenterology*, **97**, 853–859.

HOWIE, A.F., FORRESTER, L.M., GLANCEY, M.J., SCHLAGER, J.J., POWIS, G., BECKETT, G.J., HAYES, J.D. & WOLF, C.R. (1990). Glutathione S-transferase and glutathione peroxidase expression in normal and tumour human tissue. *Carcinogenesis*, **11**, 451–458.

KOSTER, A.S., RICHTER, E., LAUTERBACH, F. & HARTMANN, F. (eds) (1989). Intestinal metabolism of xenobiotics. *Prog. Pharmacol. Clin. Pharmacol.*, Vol. 7.

LASH, L.H., HAGEN, T.M. & JONES, D.P. (1986). Exogenous glutathione protects intestinal epithelial cells from oxidative injury. *Proc. Natl Acad. Sci. USA*, **83**, 4641–4645.

LOWRY, O.H., ROSEBROUGH, N.J., FARR, A.L. & RANDALL, R.J. (1951). Protein measurement with the Folin phenol reagent. *J. Biol. Chem.*, **193**, 265–275.

MANNERVIK, B. & DANIELSON, U.H. (1988). Glutathione S-transferases. Structure and catalytic activity. *CRC Crit. Rev. Biochem.*, **23**, 283–337.

MARTENSSON, J., JAIN, A., STOLE, E., FRAYER, W., AULD, P.A.M. & MEISTER, A. (1991). Inhibition of glutathione synthesis in the newborn rat: a model for endogeneously produced oxidative stress. *Proc. Natl Acad. Sci. USA*, **88**, 9360–9364.

MEYER, D.J., COLES, B., PEMBLE, S.E., GILMORE, K.S., FRASER, G.M. & KETTERER, B. (1991). Theta, a new class of glutathione transferases purified from rat and man. *Biochem. J.*, **274**, 409–414.

NOORDHOEK, J. & VAN BLADEREN, P.J. (1991). Nutrition and extrahepatic metabolism. In *Nutrition, Toxicity and Cancer*. Rowland, I.R. (ed.) pp. 93–112. CRC Press, Boca Raton, Florida.

PETERS, W.H.M., BOON, C.E.W., ROELOFS, H.M.J., WOBBES, TH., NAGENGAST, F.M. & KREMERS, P.G. (1992). Expression of drug metabolizing enzymes and P-170 glycoprotein in colorectal carcinoma and normal mucosa. *Gastroenterology*, **103**, 448–455.

PETERS, W.H.M., KOCK, L., NAGENGAST, F.M. & KREMERS, P.G. (1991). Biotransformation enzymes in human intestine: critical low levels in the colon? *Gut*, **32**, 408–412.

PETERS, W.H.M., KOCK, L. NAGENGAST, F.M. & ROELOFS, H.M.J. (1990*a*). Immunodetection with a monoclonal antibody of glutathione S-transferase mu in patients with and without carcinomas. *Biochem. Pharmacol.*, **39**, 591–597.

PETERS, W.H.M., NAGENGAST, F.M. & WOBBES, TH. (1989). Glutathione S-transferases in normal and cancerous human colon tissue. *Carcinogenesis*, **10**, 2371–2374.

PETERS, W.H.M., WORMSKAMP, N.G.M. & THIES, E. (1990*b*). Expression of glutathione S-transferases in normal gastric mucosa and in gastric tumors. *Carcinogenesis*, **11**, 1593–1596.

ROMANO, M., RAZANDI, M., RAZA, A., SZABO, S. & IVEY, J. (1992). Cysteamine protects gastric epithelial cell monolayers against drug induced damage: evidence for direct cellular protection by sulphydryl compounds. *Gut*, **33**, 30–38.

SALIM, A.S. (1990). Sulfhydryls protect patients against complications of erosive gastritis. *Digest. Dis. Sci.*, **35**, 1436–1438.

SEIDEGÅRD, J., PERO, R.W., MARKOWIK, M.M., ROUSH, G., MILLER, D.G. & BEATTIE, E.J. (1990). Isoenzymes of glutathione S-transferase (class mu) as a marker for susceptibility to lung cancer; a follow up study. *Carcinogenesis*, **11**, 33–36.

SIEGERS, C.P. (1989). Glutathione & GSH-dependent enzymes. In *Intestinal Metabolism of Xenobiotics*. Koster, A.S., Richter, E., Lauterbach, F. & Hartmann, F., (eds) *Prog. Pharmacol. Clin. Pharmacol.*, **7**, 171–180.

SPECHLER, S.J., ROBBINS, A.H., RUBINS, H.B., VINCENT, M.E., HEEREN, T., DOOS, W.G., COLTON, T. & SCHIMMEL, E.M. (1984). Adenocarcinoma and Barrett's esophagus. An overrated risk? *Gastroenterology*, **87**, 927–933.

STRANGE, R.C., MATHAROO, B., FAULDER, G.C., JONES, P., COTTON, W., ELDER, J.B. & DEAKIN, M. (1991). The human glutathione S-transferases: a case control study of the incidence of the GST 10 phenotype in patients with adenocarcinoma. *Carcinogenesis*, **12**, 25–28.

VAN OMMEN, B., BOGAARDS, J.J.P., PETERS, W.H.M., BLAAUWBOER, B. & VAN BLADEREN, P.J. (1990). Quantification of human hepatic glutathione S-transferases. *Biochem. J.*, **269**, 609–613.

VAN POPPEL, G., DE VOGEL, N., VAN BLADEREN, P.J. & DE KOK, F.J. (1992). Increased cytogenetic damage in smokers deficient in glutathione S-transferase isoenzyme mu. *Carcinogenesis*, **13**, 303–305.

WIENCKE, J.K., KELSEY, K.T., LAMELA, R.A. & TOSCANO, W.A. (1990). Human glutathione S-transferase deficiency as a marker of susceptibility to epoxide induced cytogenetic damage. *Cancer Res.*, **50**, 1585–1590.

ZHONG, S., HOWIE, A.F., KETTERER, B., TAYLOR, J., HAYES, J.D., BECKETT, G.J., WATHEN, C.G., WOLF, C.R. & SPURR, N.K. (1991). Glutathione S-transferase mu locus: use of genotype and phenotype assays to assess association with lung cancer susceptibility. *Carcinogenesis*, **12**, 1533–1537.